# Magnitude of Off-Target Allo-HLA Reactivity by Third-Party Donor-Derived Virus-Specific T Cells Is Dictated by HLA-Restriction

**DOI:** 10.3389/fimmu.2021.630440

**Published:** 2021-03-29

**Authors:** Wesley Huisman, Didier A. T. Leboux, Lieve E. van der Maarel, Lois Hageman, Derk Amsen, J. H. Frederik Falkenburg, Inge Jedema

**Affiliations:** ^1^Department of Hematology, Leiden University Medical Center, Leiden, Netherlands; ^2^Department of Hematopoiesis, Sanquin Research and Landsteiner Laboratory for Blood Cell Research, Amsterdam, Netherlands

**Keywords:** virus-specific T cells, allo-HLA cross-reactivity, adoptive T cell immunotherapy, HLA-mismatched donor, cytotoxic T cells, Graft Versus Host Disease

## Abstract

T-cell products derived from third-party donors are clinically applied, but harbor the risk of off-target toxicity *via* induction of allo-HLA cross-reactivity directed against mismatched alleles. We used third-party donor-derived virus-specific T cells as model to investigate whether virus-specificity, HLA restriction and/or HLA background can predict the risk of allo-HLA cross-reactivity. Virus-specific CD8^pos^ T cells were isolated from HLA-A^*^01:01/B^*^08:01 or HLA-A^*^02:01/B^*^07:02 positive donors. Allo-HLA cross-reactivity was tested using an EBV-LCL panel covering 116 allogeneic HLA molecules and confirmed using K562 cells retrovirally transduced with single HLA-class-I alleles of interest. HLA-B^*^08:01-restricted T cells showed the highest frequency and diversity of allo-HLA cross-reactivity, regardless of virus-specificity, which was skewed toward multiple recurrent allogeneic HLA-B molecules. Thymic selection for other HLA-B alleles significantly influenced the level of allo-HLA cross-reactivity mediated by HLA-B^*^08:01-restricted T cells. These results suggest that the degree and specificity of allo-HLA cross-reactivity by T cells follow rules. The risk of off-target toxicity after infusion of incompletely matched third-party donor-derived virus-specific T cells may be reduced by selection of T cells with a specific HLA restriction and background.

## Key Points

- HLA-restriction determines the scope of off-target reactivities.
- HLA background shapes the broadness of off-target reactivities.

## Introduction

Adoptive transfer of autologous or human leukocyte antigen (HLA)-matched patient-specific T-cell products, including antigen-specific T cells, Chimeric Antigen Receptor (CAR) T cells and T-cell receptor (TCR) modified T cells are clinically applied and show feasibility and safety (1–5). Nevertheless, the complex logistics and delays associated with the generation of these products for adoptive immunotherapy strategies are hampering easy broad application. Off-the shelf T-cell products, generated from cells of healthy third-party donors and suitable for treatment of multiple patients, may be an elegant solution, but such products are often only partially HLA-matched with the recipient.

In our study we focused on virus-specific T-cell products derived from healthy third-party donors that can be used for the treatment of uncontrolled viral reactivations and/or viral disease in patients without easy access to autologous or donor-derived virus-specific T cells. Reactivations of cytomegalovirus (CMV), Epstein-Barr virus (EBV) and adenovirus (AdV) are frequently seen and associated with high morbidity and mortality in immune-compromised patients (6, 7), like patients after allogeneic stem cell transplantation (AlloSCT), but also patients after solid organ transplantation. For patients transplanted with stem cells from a virus-non-experienced donor or in general for solid-organ donors there is no easy access to (HLA-matched) memory virus-specific T cells. Adoptive transfer of partially HLA-matched virus-specific T cells from healthy third-party donors is a potential strategy to temporarily provide anti-viral immunity to these patients. However, these third party donor-derived virus-specific T cells have not been tolerized by thymic negative selection to the non-matched HLA molecules that are present within the patient (8, 9), thereby implying the risk of off-target toxicity due to allo-HLA cross-reactivity directed against the mismatched HLA alleles (10).

It was demonstrated that third-party derived virus-specific T cells can exert allo-HLA cross-reactivity directed against mismatched HLA alleles *in vitro* (11–14). The viral specificity as well as the allo-HLA cross-reactivity was shown to be mediated by the same T-cell receptor (TCR) complex (11, 14). Additionally, TCR cross-reactivity could be a major trigger of graft rejection, as shown by the association between viral reactivation and graft rejection in recipients of solid organs (15, 16). Despite the clearly documented allo-HLA cross-reactivity of virus-specific T-cell populations documented *in vitro*, only low rates (~5%) of off-target toxicity/*de novo* Graft vs. Host Disease (GVHD) were observed in stem cell recipients that were treated with partially HLA-matched virus-specific T cells (17–21). There are several potential reasons for this discrepancy: (1) the specific allogeneic peptide/HLA complex recognized by the cross-reactive virus-specific T cells is not present in the patient, (2) removal of the *in vitro* off-target (>10% cytotoxic) virus-specific T cells from the product prior to administration to the patient and/or selection of T-cell products that do not show *in vitro* allo-HLA reactivity (18), (3) low T-cell numbers of cross-reactive virus-specific T cells administered and/or limited *in vivo* proliferation, (4) Rejection of the partly HLA-matched third party virus-specific T cells by the recipient (22). In the last example, such rejection prevents toxicity, but it also diminishes the short-term protection afforded by the third-party derived T cells. In a recent phase I/II clinical study by Neuenhahn et al., survival/persistence was only demonstrated for adoptively transferred virus-specific T cells of the original stem cell donor (8/8 HLA-matched), but not for virus-specific T cells derived from third-party donors with a higher degree of HLA-mismatch (22).

It would be useful if we could predict which non-matched HLA molecules are recognized by third-party derived T cells so that specific donors and/or specific T-cell populations can be selected with a low likelihood of exerting off-target reactivity. Thus far, recurrent off-target reactivity toward the same non-matched HLA molecule was only found for T-cell populations isolated from different individuals using the exact same TCR (public TCR) (14, 23, 24). A classic example of such public cross-reactivity is the HLA-B^*^08:01-restricted EBV-EBNA3A^FLR^-specific T-cell population that contains a dominant public TCR showing cross-reactivity against non-self HLA-B^*^44:02 (23, 24). Importantly, this public TCR is not found in the T-cell repertoire of HLA-B^*^08:01/HLA-B^*^44:02 positive individuals, demonstrating the deletion of this otherwise potentially auto-reactive public TCR during *in vivo* thymic selection. Many antiviral T-cell responses are, however, not so clearly dominated by a single dominant public TCR, making predictions of cross-reactivity more difficult.

The aim of this study was to investigate whether we could identify and predict allo-HLA cross-reactivity patterns by third-party donor-derived T cells, using virus-specific T cells as a model. We investigated whether the allo-HLA cross-reactivity by third-party donor-derived virus-specific T cells was influenced by virus-specificity, HLA-restriction and/or HLA background of the donors. Our data show that the level of allo-HLA cross-reactivity is not affected by viral-specificity, but surprisingly strongly associated with HLA restriction and influenced by the HLA background of the donors.

## Materials and Methods

### Collection of Donor Material

After informed consent according to the Declaration of Helsinki, healthy donors (homozygously) expressing HLA-A^*^01:01 and HLA-B^*^08:01 or HLA-A^*^02:01 and HLA-B^*^07:02 were selected from the Sanquin database and the biobank of the department of Hematology, Leiden University Medical Center (LUMC). Two donors expressing HLA-A^*^02:01/HLA-B^*^07:02 were not homozygous. Peripheral blood mononuclear cells (PBMCs) were isolated by standard Ficoll-Isopaque separation and used directly or thawed after cryopreservation in the vapor phase of liquid nitrogen. Donor characteristics (HLA typing, CMV and EBV serostatus) are provided in Table 1 (Donors 1–24). Healthy donors expressing HLA-B^*^08:01 and HLA-B^*^13:02 or HLA-B^*^35:01 (Table 1; donors 25–30) were selected from the biobank of the department of Hematology (LUMC).

**Table 1:** HLA typing and CMV/EBV serostatus of healthy donors.

**#**	**CMV**	**EBV**	**HLA-A**		**HLA-B**		**HLA-C**		**HLA-DR**		**HLA-DQ**		**HLA-DP**	
1	Pos	Pos	01:01		08:01		07:01		03:01	15:01	02:01	06:02	N.D	
2	Pos	Pos	01:01		08:01		07:01		03:01	01:02	02:01	05:01	01:01	04:01
3	Neg	Pos	01:01		08:01		07:01		03:XX		02:XX		N.D	
4	Pos	Pos	01:01		08:01		07:01		03:01		02:01		04:01	
5	Neg	Pos	01:01		08:01		07:01		03:01		02:01		04:01	
6	Neg	Pos	01:01		08:01		07:01		03:01		02:01		01:01	09:01
7	Pos	Pos	01:01		08:01		07:01		03:XX		02:XX		N.D	
8	Neg	Pos	01:01		08:01		07:01		03:XX		02:XX		N.D	
9	Pos	Pos	01:01		08:01		07:01		03:01		02:01		01:01	04:01
10	Neg	Pos	01:01		08:01		07:01		03:01		02:01		04:01	04:02/01
11	Neg	Pos	01:01		08:01		07:01		03:01		02:01		04:01	05:01
12	Neg	Pos	01:01		08:01		07:01		03:01		02:01		04:01	05:01
13	Pos	Pos	02:01		07:02		07:02		15:01		06:02		04:01	
14	Pos	Pos	02:01		07:02	44:02	07:02	05:01	15:01	04:01	06:02	03:01	04:XX	02:01
15	Pos	Pos	02:01	03:01	07:02		07:02		15:01		06:02		04:01	03:01
16	Pos	Pos	02:01	03:01	07:02	44:02	07:02	05:01	15:01	01:01	06:02	05:01	04:01	14:01
17	Pos	Pos	02:01		07:02		07:02		15:01		06:02		04:01	05:01
18	Pos	Pos	02:01		07:02		07:02		15:01		06:02		04:01	
19	Neg	Pos	02:01		07:02		07:02		15:01		06:02		04:01	
20	Pos	Pos	02:01		07:02		07:02		15:01		06:02		02:01	04:01
21	Neg	Pos	02:01		07:02		07:02		15:01		06:02		04:01	13:01
22	Pos	Pos	02:01		07:02		07:02		07:01	15:01	03:03	06:02	04:01	13:01
23	Neg	Pos	02:01		07:02		07:02		15:XX		06:XX		N.D	
24	Pos	Pos	02:01		07:02		07:02		15:XX		06:XX		N.D	
25	Pos	Pos	01:01	68:01	08:01	35:01	04:01	07:01	01:01	03:01	02	05:01	04:01	04:02
26	Neg	pos	01:01	24:02	08:01	35:01	04:01	07:01	03:01	08:01	02:01	04:02	04:01	
27	Pos	pos	02:01	24:02	08:01	35:01	07:01	11:01	02:02	03:01	02:02	03:01	02:01	13:01
28	Pos	pos	01:01	30:01	08:01	13:02	07:01	06:02	03:01	04:01	02:01	03:01	04:01	
29	Pos	pos	01:01	30:01	08:01	13:02	07:01	06:02	04:07	15:01	03:01	06:02	04:01	
30	Pos	pos	01:01	30:01	08:01	13:02	07:01	06:02	03:01	07:01	02:01	02:02	04:01	09:01

Virus-specific T cells restricted to HLA-A*01:01/HLA-B*08:01 or HLA-A*02:01/HLA-B*07:02 were isolated from donors ^#^1–12 and donors ^#^13–24, respectively. CMV and EBV serostatus are indicated for each donor. HLA typing was determined either by serology, where the second digits could not be determined (XX) or with high resolution HLA typing, unless indicated by N.D. HLA-B*08:01-restricted EBV-EBNA3A^QAK^ and EBV-BZLF1^RAK^-specific T cells were additionally isolated from donors ^#^25–30 for specific experiments to investigate the role of HLA-backgrounds. Blanks indicate homozygosity for the given allele.

### Isolation and Expansion of Virus-Specific T Cells

Phycoerythrin (PE), allophycocyanin (APC), BV421, BV510 and/or peridinin-chlorophyll-protein (PerCP)-labeled pMHC-tetramer complexes were used for fluorescence-activated cell sorting (FACSorting). The pMHC-tetramers used (for generation see Supplementary Material and Methods) are shown in Supplementary Table 1. PeptideMHC-tetramer positive, CD8^pos^/CD4^neg^ T cells were sorted and seeded at 10,000 cells per well in U-bottom microtiter plates for the generation of bulk T-cell populations. After 2 weeks of culture, pMHC-tetramer^pos^ T-cell populations were considered pure if they contained ≥97% pMHC-tetramer^pos^ cells. Polyclonality of the sorted virus-specific T cells was assessed by T-cell receptor-variable β (TCR-Vβ) family analysis using the TCR-Vβ kit (Beckman Coulter, Fullerton, USA). Sub-populations expressing a single TCR-Vβ family were sorted from the bulk using monoclonal antibodies from the TCR-Vβ kit. Sub-populations were then non-specifically expanded. Sub-populations using one specific TCR-Vβ family were considered pure if ≥95% of the population was positive for that TCR-Vβ family. Sorting was performed on a FACS ARIA (BD) and analyzed using Diva software (BD). All analyses were performed on a FACS Calibur (BD), and analyzed using Flowjo Software (TreeStar, Ashland, USA). Procedures to isolate and expand virus-specific T cells are described in the Supplementary Material and Methods.

### Selection and Generation of Stimulator Cells for Functional Analyses

EBV-transformed lymphoblastoid cell-lines (EBV-LCLs) were generated according to standard protocols (25). EBV-LCLs were selected to cover a total of 116 frequently occurring HLA molecules, as listed in Table 2. HLA-deficient K562 cells were transduced with 40 different single HLA-class-I molecules (Supplementary Table 2) including common and rare HLA-class-I molecules. EBV-LCLs and HLA-deficient K562 cell-lines were cultured in stimulator medium consisting of Iscove's Modified Dulbecco's Medium (IMDM; Lonza, Verviers, Belgium) supplemented with 10% heat-inactivated Fetal Bovine Serum (FBS; Invitrogen, Carlsbad, USA), 100 U/mL penicillin (Lonza), 100 μg/mL streptavidin (Lonza) and 2.7 mM L-glutamine (Lonza). Generation of various cell-lines is described in the Supplementary Material and Methods.

**Table 2:** HLA typing of the HLA-mismatched EBV-LCL panel.

**EBV-LCL**	**HLA-A**		**HLA-B**		**HLA-C**		**HLA-DR**		**HLA-DQ**		**HLA-DP**	
UBX	01:01	03:01	08:01	18:01	01:02	07:01	03:01	10:XX	02:01	05:01	01:01	02:01
ACD	01:01	24:02	39:06	51:01	07:02	14:02	01:02	11:01	03:01	05:01	04:01	04:02
GME	26:01	02:06	38:01	35:01	12:03	04:01	04:04	01:01	05:01	03:02	04:01	04:02
ABF	30:04	68:02	38:01	55:01	03:03	12:03	03:01	13:01	02:01	06:03	02:01	04:01
LSR	32:01	68:01	35:03	52:01	12:02	12:03	15:02	16:02	05:02	06:01	04:01	14:01
GML	23:01		41:01	51:01	15:02	17:01	07:01	15:01	02:01	06:02	02:01	04:01
UCE	03:01	11:01	07:02	27:05	02:02	07:02	11:01	14:54	03:01	05:03	02:01	16:01
GMS	01:01	11:01	51:01	50:01	15:02	06:02	07:01	04:07	02:01	03:01	03:01	02:01
WKD	11:01	24:02	15:02	40:01	07:02	08:01	08:03	09:XX	03:03	06:01	05:01	
UVN	03:01	11:01	14:02	35:01	04:01	08:02	01:01	13:02	05:01	06:09	05:01	10:01
MWX	01:01	34:01	15:21	35:03	04:03	12:03	01:01	15:02	05:01	06:01	06:01	13:01
GMK	01:01		07:02	57:01	06:02	07:02	04:04	13:01	03:02	06:03	04:02	15:01
MSV	03:01	33:01	07:02	14:02	07:02	08:02	01:02	04:05	03:03	05:01	02:01	04:01
CBF	02:01	11:01	35:01	44:02	04:01	05:01	03:01	09:01	02:01	03:03	01:01	04:01
AVZ	02:20	24:02	08:01	14:01	07:01	08:02	03:01	07:01	02:01	02:02	02:01	04:01
BSR	02:01	68:01	35:03	37:01	04:01	06:02	04:03	10:01	03:01	05:01	02;01	04:01
RHP	03:01	31:01	07:02	40:01	03:04	07:02	13:02	15:01	06:02	06:04	04:01	13:01
SOM	02:60	23:01	15:10	57:03	03:04	18:02	11:01	13:01	03:01	05:01	04:02	40:01
UBM	03:01	24:02	15:01	44:03	03:04	16:01	04:01	07:01	02:02	03:02	03:01	
UBG	02:01	30:02	15:01	39:01	03:03	12:03	01:01	13:01	05:04	06:03	02:01	04:01
LMB	29:02		44:03	51:01	14:02	16:01	07:01	08:01	02:02	04:02	04:01	11:01
FAQ	23:01	68:02	14:02	38:01	08:02	12:03	13:01	13:03	03:01	06:03	02:01	
OBB	01:01	02:01	07:02	08:01	07:01	07:02	03:01	15:01	02:01	06:02	01:01	05:01

*The panel of HLA-mismatched EBV-LCLs was composed of high resolution HLA-typed EBV-LCLs that together covered all HLA-class-I and almost all frequently HLA-class-II molecules that are frequently (2%) occurring in the Caucasian population. The HLA typing was determined molecularly. XX indicates that only the allele group could be determined (2 digit resolution). Blanks indicate homozygosity for the given allele*.

### Cytokine Production Assays to Determine T-Cell Reactivity

Interferon-γ (IFN-γ) production by virus-specific T cells was quantified using standard enzyme-linked immunosorbent assays (ELISA) according to the manufacturer's instructions (Sanquin Reagents, The Netherlands). Responder T cells were co-cultured with stimulator cells at a ratio of 1:10 (responder: stimulator) for 16 h at 37°C in T-cell medium used for expansion of T-cell populations as described in Supplementary Material using 25 IU/ml Interleukin-2 (IL-2) instead of 100 IU/ml IL-2. Recognition of HLA-mismatched EBV-LCLs, HLA-matched peptide-pulsed EBV-LCLs and K562 cells transduced with specific HLA molecules was defined as production of ≥200 pg/ml of IFN-γ.

### Statistical Analysis

Statistical analyses were only performed on quantitative data and were performed using non-parametric tests. The Fisher's-Exact-test was used to assess the differences in cross-reactivity (present or absent) of HLA-mismatched EBV-LCLs between groups (i.e., HLA-A^*^01:01- and HLA-B^*^08:01-restricted virus-specific T cells). Differences in the numbers of recognized HLA-mismatched EBV-LCLs was first assessed by the Kruskal-Wallis test. Differences between two groups were then further assessed with the non-parametric Mann-Whitney U test. *p*-values were adjusted by the Bonferroni correction for multiple testing. Statistical analyses were conducted using GraphPad Prism (GraphPad Software, version 8).

## Results

### Virus-Specific T-Cell Populations Show Profound and Diverse Cross-Reactivity Against a Panel of HLA-Mismatched EBV-LCLs

To study the influence of HLA restriction and antigen specificity on the level of allo-HLA cross-reactivity mediated by virus-specific T cells, bulk virus-specific T-cell populations targeting single epitopes were isolated from total PBMCs of(homozygous) HLA-A^*^01:01/HLA-B^*^08:01^pos^ or HLA-A^*^02:01/HLA-B^*^07:02^pos^ healthy donors. Two donors did not homozygously express HLA-A^*^02:01/HLA-B^*^07:02. All donors were EBV seropositive and 5 out of 12 HLA-A^*^01:01/HLA-B^*^08:01^pos^ donors and 9 out of 12 HLA-A^*^02:01/HLA-B^*^07:02^pos^ donors were CMV seropositive (Table 1). The serostatus for AdV was unknown for all donors. Virus-specific T cells were isolated by FACS using pMHC-tetramers for various peptides (*n* = 21) from CMV, EBV, and AdV (Supplementary Table 1). In total, 45 CMV, 95 EBV and 24 AdV-specific T-cell populations were isolated (Table 3). CMV and EBV-specific T-cell populations could be isolated from all CMV^pos^ and EBV^pos^ donors, respectively. Although no AdV serostatus was known, AdV-specific T-cell populations could be isolated from 18 out of 24 donors. We isolated 22 different HLA-A^*^01:01-restricted virus-specific T-cell populations, 69 HLA-A^*^02:01-restricted virus-specific T-cell populations, 34 HLA-B^*^07:02-restricted virus-specific T-cell populations and 39 HLA-B^*^08:01-restricted virus-specific T-cell populations (Table 3). These T-cell populations were analyzed for allo-HLA cross-reactivity using a panel of HLA-mismatched EBV-LCLs, expressing the most frequent (>2%) HLA-class-I and class-II antigens in the Caucasian population (Table 2). EBV-specific T-cell populations were only tested against HLA-mismatched EBV-LCLs that did not express the specific restriction molecules to avoid recognition of EBV-derived peptides in self-HLA. In total, 65 out of 164 (39%) virus-specific T-cell populations produced interferon-γ in response to stimulation with at least one HLA-mismatched EBV-LCL (Supplementary Figure 1A).

**Table 3:** Isolated virus-specific T-cell populations.

				**Number of isolated T-cell populations/maximum** **number of isolations (%)**		
**Virus**	**Antigen**	**HLA**	**Peptide**	**Per specificity**	**Per virus**	**Per HLA**
CMV	pp50	HLA-A*01:01	VTEHDTLLY	4/5 (80%)	**CMV:** 45/56 (80.3%)	HLA-A*01:01: 22/28 (78.6%)
	pp65	HLA-A*01:01	YSEHPTFTSQY	4/5 (80%)		
	pp65	HLA-A*02:01	NLVPMVATV	7/9 (78%)		
	IE-1	HLA-A*02:01	VLEETSVML	5/9 (56%)		
	pp65	HLA-B*07:02	TPRVTGGGAM	8/9 (89%)		
	pp65	HLA-B*07:02	RPHERNGFTVL	8/9 (89%)		HLA-A*02:01: 69/90 (76.7%)
	IE-1	HLA-B*08:01	ELRRKMMYM	5/5 (100%)		
	IE-1	HLA-B*08:01	QIKVRVDMV	4/5 (80%)		
EBV	LMP2	HLA-A*01:01	ESEERPPTPY	5/6 (83%)	**EBV:** 95/114 (83.3%)	
	LMP2	HLA-A*02:01	FLYALALLL	11/12 (92%)		
	LMP2	HLA-A*02:01	CLGGLLTMV	9/12 (75%)		
	EBNA3C	HLA-A*02:01	LLDFVRFMGV	7/12 (58%)		HLA-B*07:02: 34/42 (81%)
	BMLF1	HLA-A*02:01	GLCTLVAML	10/12 (83%)		
	BRLF1	HLA-A*02:01	YVLDHLIVV	12/12 (100%)		
	EBNA3A	HLA-B*07:02	RPPIFIRRL	11/12 (92%)		
	BZLF1	HLA-B*08:01	RAKFKQLL	9/12 (75%)		
	EBNA3A	HLA-B*08:01	FLRGRAYGL	10/12 (83%)		HLA-B*08:01: 39/46 (84.8%)
	EBNA3A	HLA-B*08:01	QAKWRLQTL	11/12 (92%)		
AdV	HEXON	HLA-A*01:01	TDLGQNLLY	9/12 (75%)	**AdV:** 24/36 (66.7%)	
	E1A	HLA-A*02:01	LLDQLIEEV	8/12 (67%)		
	HEXON	HLA-B*07:02	KPYSGTAYNAL	7/12 (58%)		

Twelve donors were used to isolate HLA-A*01:01/B*08:01-restricted virus-specific T-cell populations and 12 donors were used to isolate HLA-A*02:01/B*07:02-restricted virus-specific T-cell populations. Five out of 12 HLA-A*01:01/B^*^08:01^pos^ donors were seropositive for CMV and 9 out of 12 HLA-A*02:01/B*07:02^pos^ donors were seropositive for CMV.

*CMV, Cytomegalovirus; EBV, Epstein-Barr virus; AdV, Adenovirus*.

Next, we investigated whether the T-cell populations that did not recognize any HLA-mismatched EBV-LCL contained smaller sub-population(s) of T cells that could recognize HLA-mismatched EBV-LCLs, but were missed in the initial bulk analysis. Sub-populations were sorted based on expression of single TCR-Vβ families. Twenty-four different TCR-Vβ families can be identified with the provided monoclonal antibodies in the kit that was used for flow cytometry, covering around 70% of the human TCR-Vβ repertoire (26). Sub-populations that could not be stained with the antibody kit could not be separated from the bulk populations using this strategy and were not analyzed for recognition of HLA-mismatched EBV-LCLs. In total, 165 sub-populations expressing a single TCR-Vβ family were isolated from the 99 bulk T-cell populations that initially did not show reactivity against the EBV-LCL panel. These sub-populations were subsequently analyzed for their capacity to recognize HLA-mismatched EBV-LCLs. We observed that 31 of these isolated sub-populations contained T cells that were capable of exerting allo-HLA cross-reactivity (Supplementary Figure 1B). Additionally, 193 sub-populations were sorted from bulk T-cell populations that did already demonstrate HLA-mismatched EBV-LCL recognition in the initial analysis (derived from 65 initial bulk populations). Eighty-six of these isolated sub-populations contained T cells that recognized HLA-mismatched EBV-LCLs. Recognition of additional HLA-mismatched EBV-LCLs could be observed that were not detected in the initial analysis of 25 different bulk T-cell populations (Supplementary Figure 2). In summary, a total of 83 bulk T-cell populations contained T cells that showed detectable cross-reactivity against one or more HLA-mismatched EBV-LCL(s).

**Figure 1:**
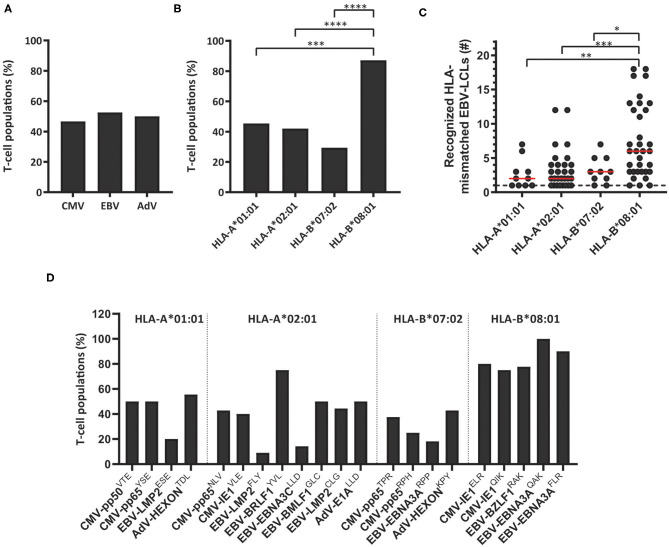
The scope of HLA-mismatched EBV-LCL recognition by virus-specific T-cell populations. Virus-specific T-cell populations (*n* = 164) were stimulated with a panel of HLA-mismatched EBV-LCLs for 16 h and IFNγ production was measured by ELISA. EBV-specific T-cell populations were tested only against those HLA-mismatched EBV-LCLs that did not express the specific restriction molecule of the viral specificity of those T cells. In total, 83 virus-specific T-cell populations contained T cells that showed recognition of HLA-mismatched EBV-LCLs, defined as production of >200 pg IFNγ/ml. **(A,B)** Shown are the frequencies of virus-specific T-cell populations that recognized HLA-mismatched EBV-LCLs per virus-specificity **(A)** and per HLA-restriction of the viral specificity **(B)**. **(C)** The number of recognized HLA-mismatched EBV-LCLs is shown for the 83 virus-specific T-cell populations that contained T cells that showed cross-reactivity against one or more HLA-mismatched EBV-LCLs (117 sub-populations were included). Recognition of the same HLA-mismatched EBV-LCL by the bulk T-cell populations and sub-population(s) derived from those initial populations was counted once. **(D)** Shown are frequencies of virus-specific T-cell populations recognizing HLA-mismatched EBV-LCLs for each viral specificity. Statistical differences were assessed with the Chi-Squared Fishers Exact-Test (A/B) or the Mann-Whitney *t-*test **(C)**. *P < 0.05; **P < 0.01; ***P < 0.001; ****P < 0.0001. Red lines represent medians. AdV, Adenovirus; EBV, Epstein-Barr virus; CMV, Cytomegalovirus.

These 83 T-cell populations were used to investigate whether the virus specificity (CMV, EBV or AdV) or HLA restriction (HLA-A^*^01:01, HLA-A^*^02:01, HLA-B^*^07:02 or HLA-B^*^08:01) of the virus-specific reactivity influences the occurrence and frequency of HLA-mismatched EBV-LCL recognition. A similar proportion of the virus-specific T-cell populations targeting CMV, EBV or AdV exerted reactivity against at least one HLA-mismatched EBV-LCL (Figure 1A). In contrast, a significantly larger fraction of the HLA-B^*^08:01-restricted virus-specific T-cell populations showed recognition of HLA-mismatched EBV-LCLs, as compared to the HLA-A^*^01:01, HLA-A^*^02:01, and HLA-B^*^07:02-restricted virus-specific T-cell populations (Figure 1B). To assess the broadness of the cross-reactivity patterns, we counted how many different HLA-mismatched EBV-LCLs were recognized by the individual T-cell populations (65 bulk T-cell populations including 86 additional sub-populations derived from these T-cell populations, and 31 sub-populations derived from the 18 bulk T-cell populations that did not recognize any HLA-mismatched EBV-LCLs in the initial analysis). In these analyses, HLA-B^*^08:01-restricted virus-specific T cells exhibited a significantly broader cross-reactivity pattern, illustrated by reactivity against a median of 6 different HLA-mismatched EBV-LCLs, whereas HLA-A^*^01:01, HLA-A^*^02:01 and HLA-B^*^07:02-restricted virus-specific T-cell populations showed reactivity against a median of only 2, 2 and 3 HLA-mismatched EBV-LCLs, respectively (Figure 1C). Within the different HLA-B^*^08:01-restricted T-cell populations, similar high frequencies of T cells capable of exerting cross-reactivity against HLA-mismatched EBV-LCLs were observed, regardless of viral antigen-specificity (Figure 1D). These results show that the occurrence and frequency of cross-reactivity against HLA-mismatched EBV-LCLs is highly affected by HLA-restriction and not by virus-specificity (CMV, EBV or AdV).

### Cross-Reactivity Against HLA-Mismatched EBV-LCLs Is Mediated by Recognition of Allogeneic HLA Molecules

**Figure 2:**
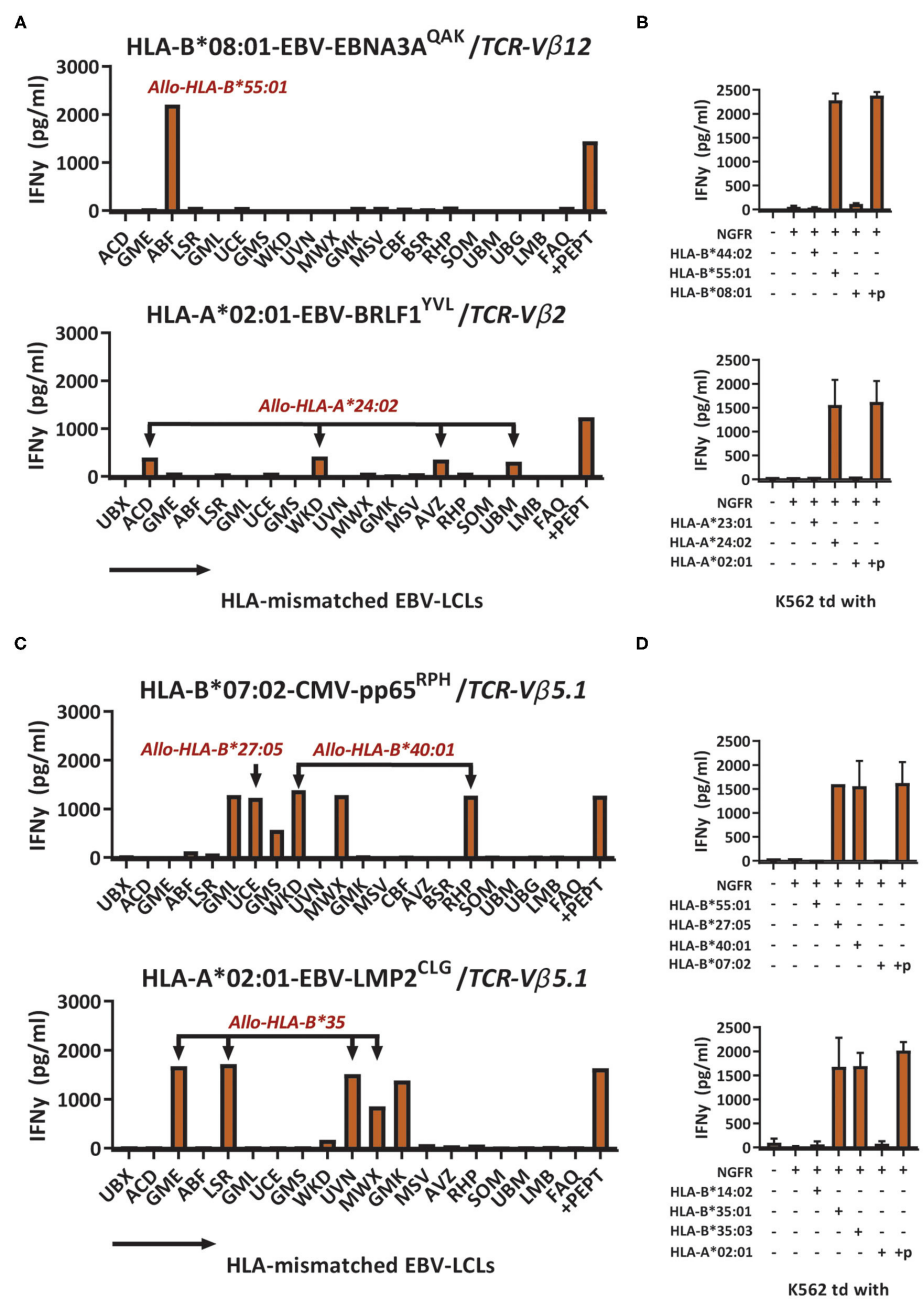
Cross-reactivity against HLA-mismatched EBV-LCLs is mediated by recognition of allogeneic HLA molecules. Sub-populations expressing a single TCR-Vβ family were sorted from the bulk virus-specific T-cell populations targeting a single epitope and were stimulated with a panel of HLA-mismatched EBV-LCLs for 16 h and IFNγ production was measured by ELISA. EBV-specific T-cell populations were tested only against those HLA-mismatched EBV-LCLs that did not express the specific restriction molecule of the viral reactivity of those T cells. Reactivity was defined as production of >200 pg/ml IFNγ. **(A)** Shown are two representative examples of virus-specific T-cell sub-populations that showed production of IFNγ (y-axis) in response to stimulation with HLA-mismatched EBV-LCLs (x-axis). **(B)** EBV-EBNA3A^QAK^ and EBV-BRLF1^YVL^-specific T cells were stimulated with K562 cells transduced with the HLA molecules that were expected to be recognized based on the patterns of reactivity against the EBV-LCL panel. K562 cells were also transduced with HLA molecules that were not expected to be recognized as negative control. K562 cells transduced with HLA-A*02:01 or HLA-B*08:01 exogenously loaded with 10^−6^M of the respective viral peptide were used as positive control. **(C)** Shown are two representative examples of virus-specific T-cell sub-populations that showed production of IFNγ (y-axis) in response to stimulation with HLA-mismatched EBV-LCLs (x-axis) that shared multiple different allogeneic HLA molecules. **(D)** CMV-pp65^RPH^ and EBVLMP2^CLG^-specific T-cell populations were stimulated with K562 cells transduced with HLA molecules that were expected to be recognized based on the reactivities seen against the EBV-LCL panel. K562 cells transduced with HLA-B*07:02 or HLA-B*08:01 exogenously loaded with 10^−6^M of the respective viral peptide were used as positive control. TCR, T-cell Receptor. Vβ, Variable Beta Chain. +*p*, peptide pulsed; NGFR, Nerve Growth Factor Receptor; td, transduced.

To investigate if the recognition of HLA-mismatched EBV-LCLs was indeed caused by recognition of allogeneic HLA molecules, HLA-deficient EBV^neg^ K562 cell-lines transduced with single HLA-class-I molecules were used as stimulator cells (12, 27). T-cell populations exhibiting a clear pattern of EBV-LCL recognition, corresponding with the expression of a single HLA allele, were tested against K562 cells transduced with the respective HLA-molecule. For example, a population of EBV-EBNA3A^QAK^-specific T cells recognized HLA-mismatched EBV-LCL ABF, which uniquely expressed HLA-A^*^30:04 and HLA-B^*^55:01 (Figure 2A). Recognition of K562 cells that were transduced with HLA-B^*^55:01 confirmed part of this respective cross-reactivity pattern (Figure 2B). In another example, a population of EBV-BRLF1^YVL^-specific T cells recognized HLA-mismatched EBV-LCLs ACD, WKD, AVZ and UBM that all expressed HLA-A^*^24:02 (Figure 2A) and this was confirmed by recognition of K562 cells transduced with HLA-A^*^24:02 (Figure 2B). Some virus-specific T-cell populations (especially HLA-B^*^08:01-restricted T cells) showed more complex reactivity patterns when tested against the EBV-LCL panel, that could not be (fully) attributed to recognition of a single allogeneic HLA-molecule. The first representative example shows CMV-pp65^RPH^-specific T cells that recognized multiple different EBV-LCLs, not allowing direct complete elucidation of the HLA allele(s) being recognized (Figure 2C). Only part of the reactivity could be explained by the unique shared expression of HLA-B^*^40:01 in EBV-LCLs WKD and RHP, that were both recognized. EBV-LCL UCE was the only EBV-LCL expressing HLA-B^*^27:05. However, the HLA alleles underlying the recognition of EBV-LCLs GML, GMS, and MWX could not be deduced. Similarly, EBV-LMP2^CLG^-specific T cells recognized 4 EBV-LCLs with unique shared expression of HLA-B^*^35:01 or HLA-B^*^35:03, while the recognition of EBV-LCL GMK could not be traced back to a specific HLA allele (Figure 2C and Supplementary Figure 3). Recognition of the HLA molecules that were anticipated to partly underlie the cross-reactivity patterns was confirmed using K562 cells transduced with the respective HLA molecules (Figure 2D). No recognition was observed of K562 cells transduced with irrelevant HLA molecules, whereas recognition of K562 cells transduced with the HLA restriction molecule of the respective virus-specific T-cell population only occurred upon exogenous peptide loading (Figures 2B,D). Allo-HLA cross-reactive virus-specific T cells also showed to be able to lyse HLA-mismatched target cells (Supplementary Figure 4), in line with previous studies (11, 14). These results show that recognition of HLA-mismatched EBV-LCLs can be mediated by recognition of single or multiple allogeneic HLA-molecules.

### HLA-B^*^08:01-Restricted Virus-Specific T Cells Recognize Multiple Allogeneic HLA Molecules, Skewed Toward Recognition of HLA-B Alleles

**Figure 3:**
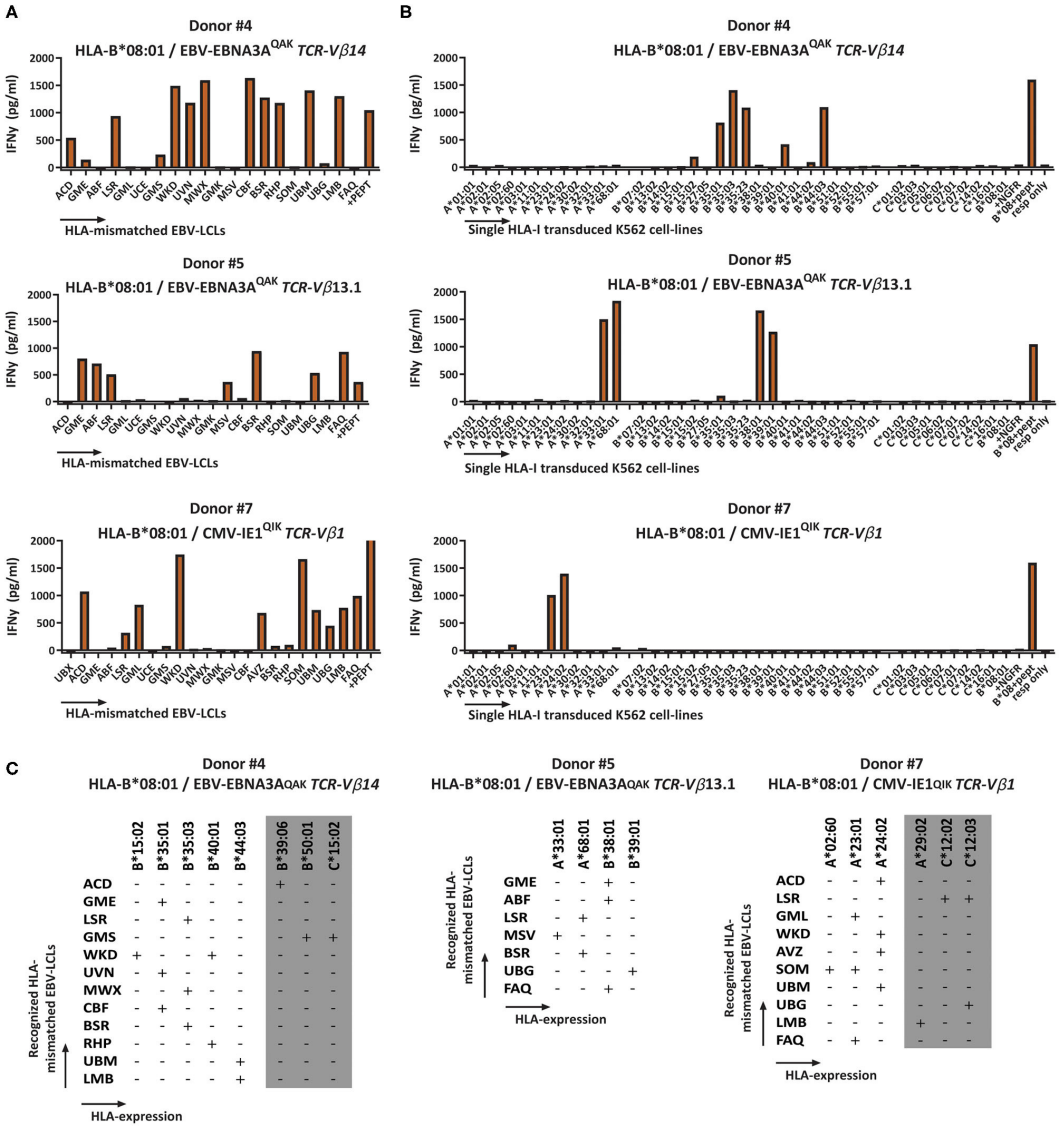
Recognition of multiple HLA-mismatched EBV-LCLs and multiple allogeneic HLA molecules by virus-specific T-cell populations. HLA-B*08:01-restricted T-cell populations that showed reactivity against multiple HLA-mismatched EBV-LCLs were subsequently tested against a panel of single HLA-class-I-transduced K562 cell-lines (*n* = 40) and IFNγ production was measured by ELISA after 16 h to analyze which specific HLA molecules were being recognized. Reactivity was defined as production of >200 pg/ml IFNγ. The K562 panel covered 63% of HLA-A, 73% of HLA-B and 37% of the HLA-C molecules that were present in the EBV-LCL panel. **(A)** Shown are three representative examples of HLA-B*08:01-restricted virus-specific T-cell populations, sorted for expression of a single TCR-Vβ family tested for production of IFNγ (y-axis) in response to stimulation with a panel of HLA mismatched EBV-LCLs (x-axis) **(B)** A panel of single HLA-class-I transduced K562 cell-lines allowed partial deduction of the recognized allogeneic HLA molecules. **(C)** Listed are the EBV-LCLs that were recognized by the respective virus-specific T-cell populations. The reactivity pattern observed with the EBV-LCL panel could partly be explained by recognition of specific HLA-class-I alleles confirmed with the K562 panel. However, some EBV-LCLs did not express any of the HLA molecules present in the K562 panel. Their recognition might be explained by recognition of HLA molecules that were not present in our K562 panel (gray). TCR, T-cell Receptor; Vβ, Variable Beta Chain; NGFR, Nerve growth factor receptor; Resp, responder.

The cross-reactivity patterns against the EBV-LCL panel of more than half of the HLA-B^*^08:01-restricted T-cell populations were rather complex and extensive (observed in 11 out of the 12 HLA-B^*^08:01^pos^ donors), even when the complexity of the T-cell populations was reduced by selecting for cells expressing a single TCR-Vβ family (Representative examples; Figure 3A). No correlation could be observed for recognition of EBV-LCLs that show shared expression of specific HLA-class-II molecules. To investigate whether the reactivity patterns of these HLA-B^*^08:01-restricted T-cell populations could be (fully) attributed to recognition of a limited number of allogeneic HLA-class-I alleles, a panel of 40 different single HLA-class-I-transduced K562 cell-lines was used as stimulator cells (Supplementary Table 2). With this panel we covered 63% of the HLA-A, 73% of the HLA-B and 37% of the HLA-C alleles present in our EBV-LCL panel. Testing the HLA-B^*^08:01-restricted T-cell populations against this K562 panel revealed recognition of multiple specific groups of allogeneic HLA alleles by single T-cell populations, which could in part explain the cross-reactivity patterns observed when tested against the EBV-LCL panel (Figures 3B,C).

**Figure 4:**
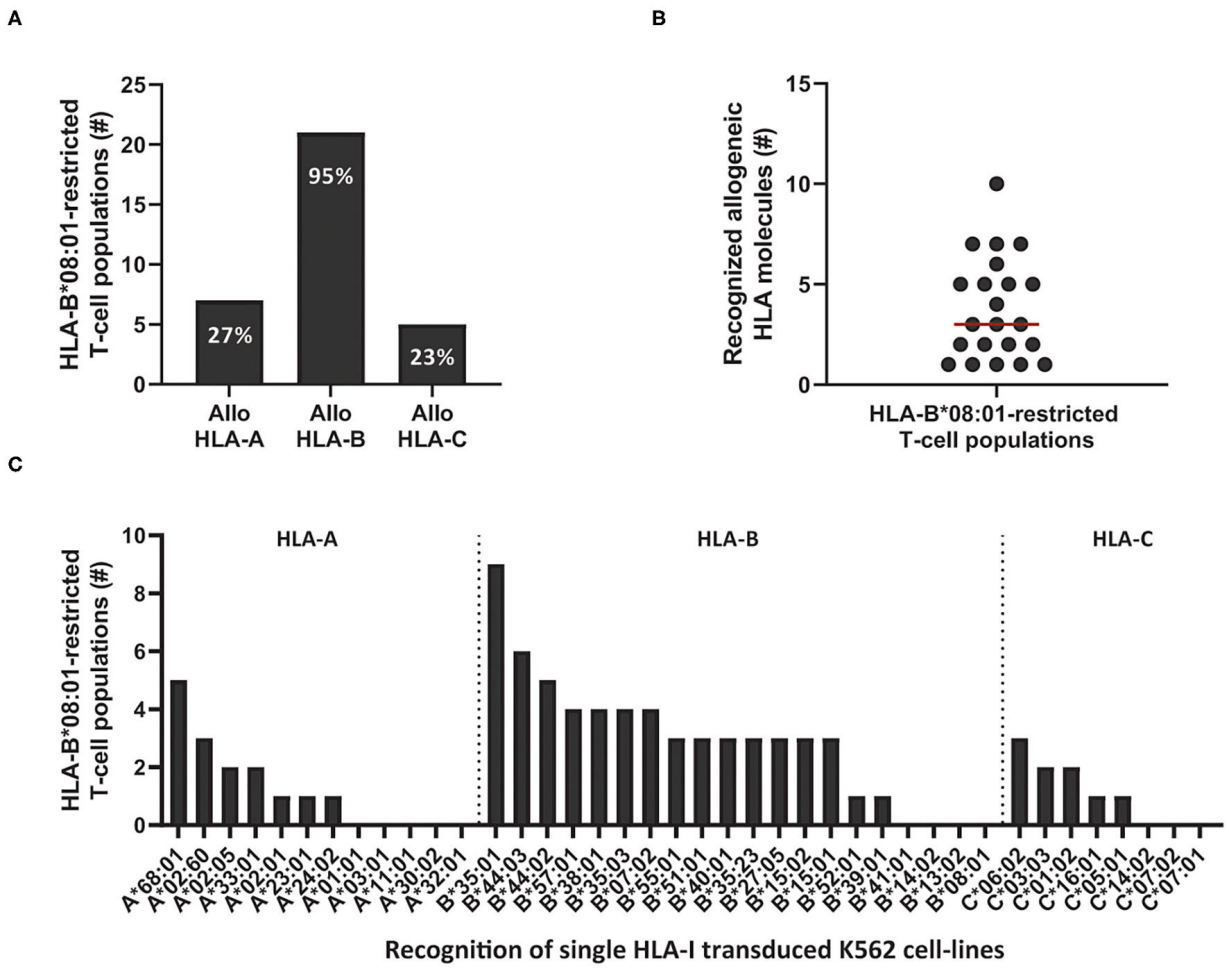
Cross-reactivity by HLA-B*08:01-restricted virus-specific T cells is skewed toward recognition of certain allogeneic HLA-B alleles. Eleven out of 12 HLA-B*08:01^pos^ donors contained HLA-B*08:01-restricted T-cell populations (*n* = 22) with no clear recognition pattern when tested against the HLA-mismatched EBV-LCL panel. To analyze which HLA molecules were being recognized, virus-specific T-cell populations, were stimulated with a panel of single HLA-class-I-transduced K562 cell-lines (*n* = 40) for 16 h and IFNγ production was measured by ELISA. Reactivity was defined as production of >200 pg/ml IFNγ. **(A)** Shown are the number of HLA-B*08:01-restricted T-cell populations that contained T cells that recognized allogeneic HLA-A, B or C alleles. Some populations were allocated to multiple groups. **(B)** Shown are the number of recognized allogeneic HLA molecules for each HLA-B*08:01-restricted T-cell population. Red line represents median **(C)** Shown are the numbers of HLA-B*08:01-restricted T-cell populations (y-axis) that show recognition of specific allogeneic HLA-A, B or C alleles (x-axis).

Next, we determined if the cross-reactivity of HLA-B^*^08:01-restricted T-cell populations was skewed toward HLA-A, B, or C molecules. In total, 22 HLA-B^*^08:01-restricted bulk or sub-populations (derived from the 11 HLA-B^*^08:01^pos^ donors that contained complex and extensive cross-reactive virus-specific T-cell populations) were tested against the K562 panel expressing a selection of HLA-A, B, and C alleles. Twenty-one out of 22 HLA-B^*^08:01-restricted T-cell populations recognized at least one allogeneic HLA-B molecule and 1 HLA-B^*^08:01-restricted T-cell population (CMV-IE1^QIK^ from donor 7) only recognized multiple HLA-A molecules in this panel (Figure 4A and Supplementary Figure 5). Twelve out of 21 allo-HLA-B-reactive HLA-B^*^08:01-restricted T-cell populations recognized only allogeneic HLA-B molecules and 9 T-cell populations additionally recognized allogeneic HLA-A and/or HLA-C molecules (Supplementary Figure 5). The number of allogeneic HLA-class-I molecules in the K562 panel recognized by the 22 HLA-B^*^08:01-restricted virus-specific T-cell populations ranged from 1 to 10 per T-cell population (median of 3; Figure 4B). HLA-B^*^35:01, B^*^44:02 and B^*^44:03 were most frequently recognized, whereas HLA-B^*^13:02, HLA-B^*^14:02 and HLA-B^*^41:01 were never recognized by HLA-B^*^08:01-restricted T cells (Figure 4C).

**Figure 5:**
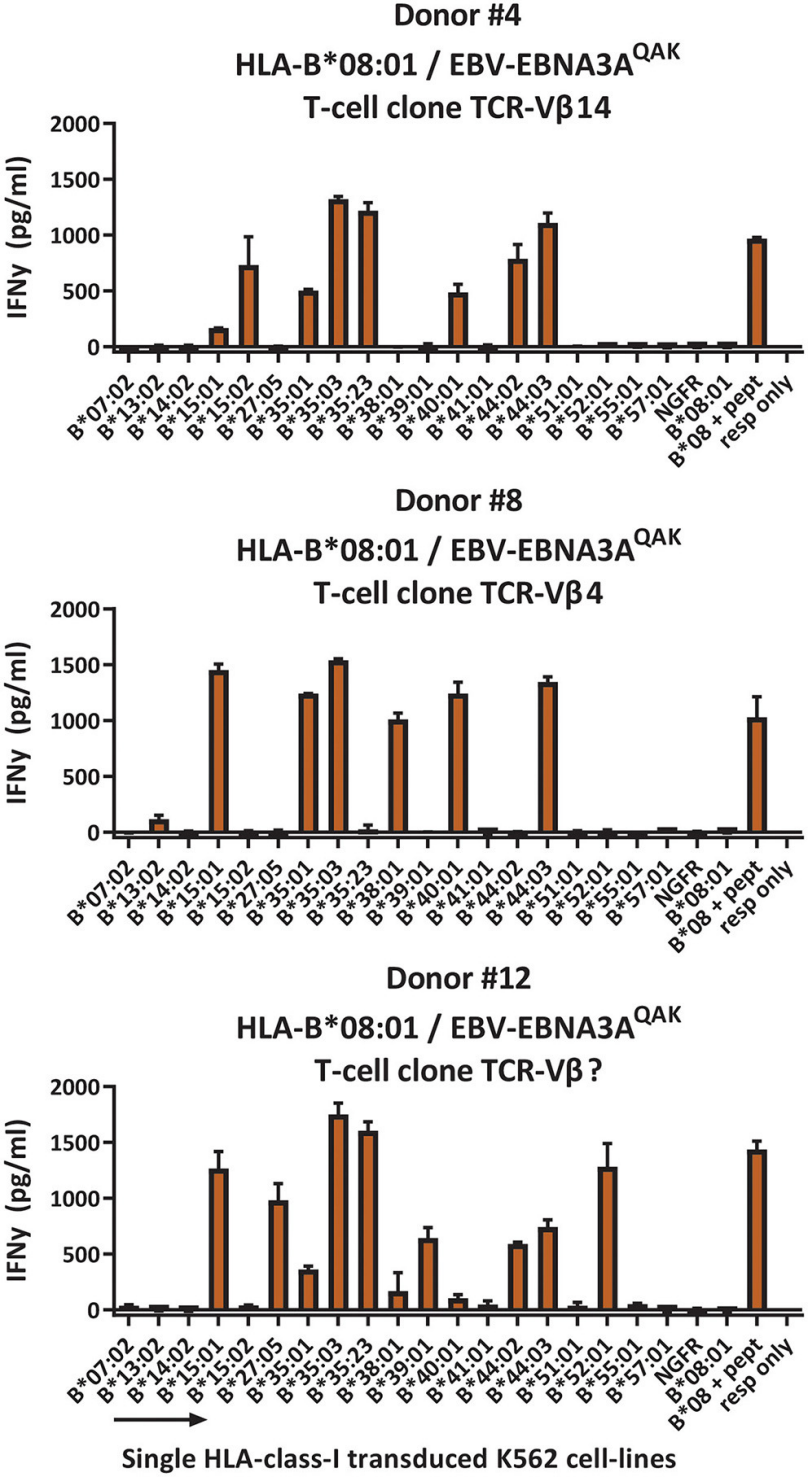
HLA-B*08:01-restricted EBV-EBNA3A^QAK^-specific T-cell clones recognize multiple allogeneic HLA molecules. HLA-B*08:01-restricted EBV-EBNA3A^QAK^-specific T-cell clones showing cross-reactivity against allogeneic HLA-B*35:01 were sorted from EBV-EBNA3A^QAK^-tetramer positive bulk T-cell populations based on expression of activation marker CD137 after stimulation with K562 cells transduced with HLA-B*35:01. All virus-specific T-cell clones showed 100% positive EBV-EBNA3A^QAK^-tetramer staining. Six T-cell clones per donor were stimulated with a panel of single HLA-B molecule-transduced K562-cell lines (x-axis) for 16 h and IFNγ production (y-axis) was measured by ELISA to analyze which HLA molecules were being recognized. Reactivity was defined as production of >200 pg/ml IFNγ. One representative T-cell clone is shown for each donor. T-cell clones from donor #4 expressed TCR-Vβ14, T-cell clones from donor #8 expressed TCR-Vβ4 and T-cell clones from donor #12 expressed a TCR-Vβ family that could not be determined by the TCR-Vβ flow cytometry kit. Shown are means with standard deviations of 1 experiment carried out in triplicate. TCR, T-cell Receptor; Vβ, Variable Beta Chain; NGFR, Nerve growth factor receptor; Resp, responder.

To investigate whether the complex and extensive recognition of allogeneic HLA molecules could be mediated by one T-cell clone, we generated T-cell clones from three cross-reactive EBV-EBNA3A^QAK^-specific T-cell populations (donor #4; donor #8 and #12; Supplementary Figure 5). Indeed, all T-cell clones recognized multiple HLA-B alleles in the K562 panel, in the same pattern as the initial EBV-EBNA3A^QAK^-specific T-cell populations (1 representative example per donor; Figure 5), demonstrating that single T-cell clones can exert complex and extensive cross-reactivity against allogeneic HLA molecules. Although these T-cell clones expressed different TCR-Vβ families, all T-cell clones showed a recurrent pattern of recognition of both HLA-B^*^15:01, HLA-B^*^35:01, HLA-B^*^35:03, HLA-B^*^40:01, and HLA-B^*^44:03.

### The HLA Background of Donors Shapes the Allo-HLA Cross-Reactivity of HLA-B^*^08:01-Restricted T Cells

**Figure 6:**
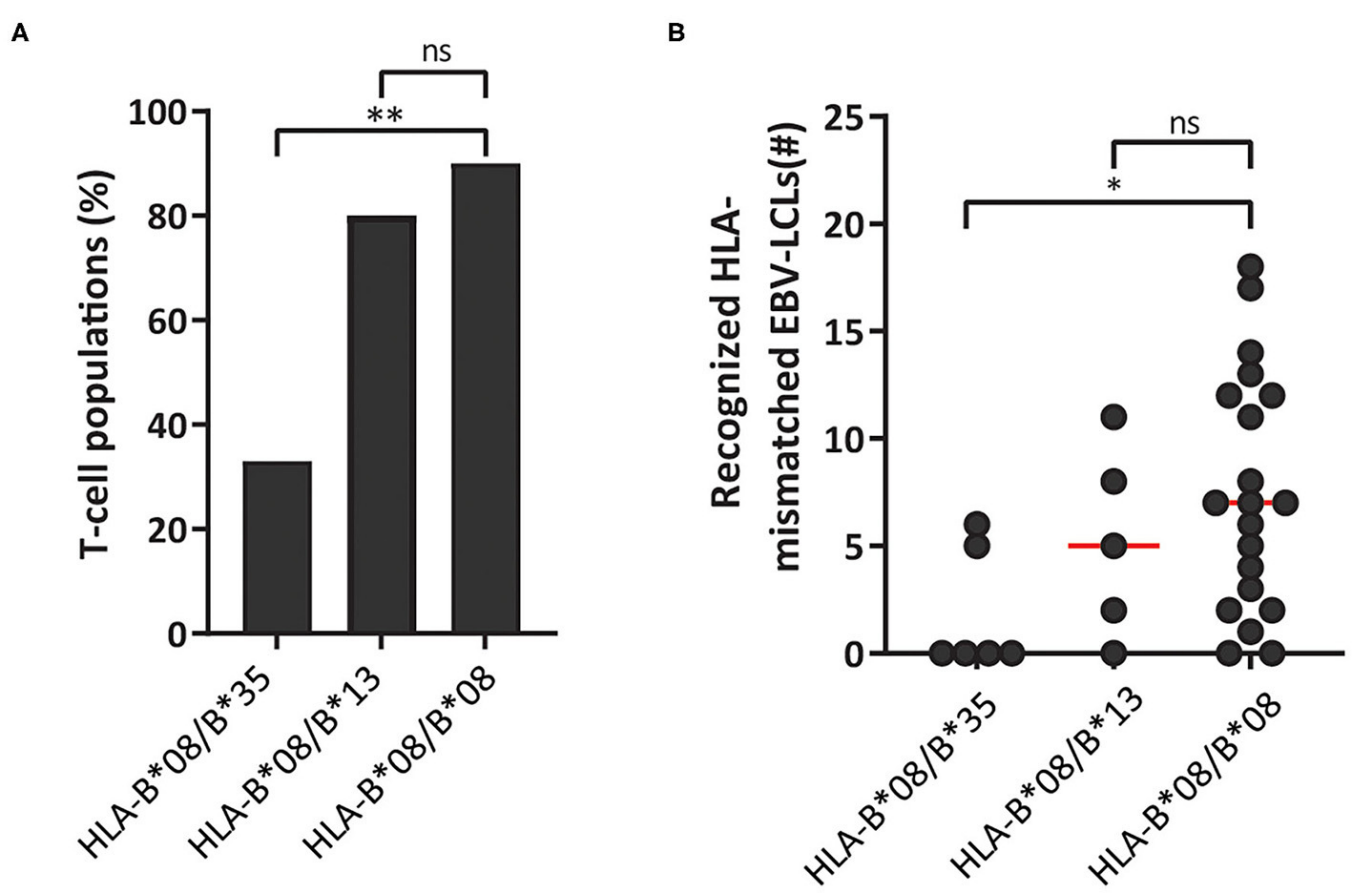
HLA-background of donors shapes the allo-HLA cross-reactivity of HLA-B*08:01-restricted T cells. HLA-B*08:01-restricted EBV-EBNA3A^QAK^ and EBV-BZLF1^RAK^-specific T-cell populations isolated from HLA-B*08:01/B*35:01^pos^ donors, HLA-B*08:01/B*13:02^pos^ donors or HLA-B*08:01 homozygous donors were stimulated with a panel of HLA-mismatched EBV-LCLs for 16 h and IFNγ production was measured by ELISA. Reactivity was defined as production of >200 pg/ml IFNγ. EBV-LCLs expressing the HLA restriction molecules of the viral specificity of the respective T-cell populations were excluded. Sub-populations of T cells expressing single TCR-Vβ families derived from the initial HLA-B*08:01-restricted EBV-EBNA3A^QAK^ and EBV-BZLF1^RAK^-specific T-cell populations were included in the analysis of the level of reactivity against the HLA-mismatched EBV-LCL panel. **(A)** The percentages of the HLA-B*08:01-restricted EBV-BZLF1^RAK^ and EBV-EBNA3A^QAK^-specific T-cell populations isolated from HLA-B*08:01/B*35:01^pos^ donors, HLA-B*08:01/B*13:02^pos^ donors or HLA-B*08:01 homozygous donors that recognize 1 or more HLA-mismatched EBV-LCLs were compared. **(B)** Shown are the number of HLA-mismatched EBV-LCLs (y-axis) that are recognized by HLA-B*08:01-restricted EBV-EBNA3A^QAK^ and EBV-BZLF1^RAK^-specific T-cell populations isolated from HLA-B*08:01/B*35:01^pos^, HLA-B*08:01/B*13:02^pos^ or HLA-B*08:01^pos^ homozygous donors. Statistical differences were assessed with a Chi-Squared Fishers Exact Test **(A)** and the Mann-Whitney *t-*test **(B)**. *P < 0.05; **P < 0.01. Red lines represent medians.

Virus-specific T cells from HLA-B^*^08:01 homozygous donors frequently recognized HLA-B^*^35:01. We therefore reasoned that heterozygosity for HLA-B^*^35:01 would purge much of the cross-reactivity from the HLA-B^*^08:01-restricted TCR-repertoire through thymic negative selection. For these reasons, HLA-B^*^08:01-restricted EBV-EBNA3A^QAK^ and EBV-BZLF1^RAK^-specific T-cell populations (*n* = 35) were isolated from 3 HLA-B^*^08:01/HLA-B^*^35:01^pos^ heterozygous donors (Table 1). Contrary, HLA-B^*^13:02 was never recognized by T cells from HLA-B^*^08:01^pos^ donors. Therefore, we also isolated T-cell populations (*n* = 10) with the same specificities from 3 HLA-B^*^08:01^pos^ donors, heterozygous for HLA-B^*^13:02 (Table 1). Strikingly, only 33% of the T-cell populations isolated from HLA-B^*^08:01/B^*^35:01^pos^ heterozygous donors recognized one or more HLA-mismatched EBV-LCLs, while 90% of the corresponding T-cell populations from HLA-B^*^08:01^pos^ homozygous donors demonstrated recognition of HLA-mismatched EBV-LCLs (Figure 6A). In contrast, 80% of the T-cell populations isolated from HLA-B^*^08:01/HLA-B^*^13:02 heterozygous donors recognized one or more HLA-mismatched EBV-LCLs (Figure 6A). HLA-B^*^08:01-restricted T cells isolated from HLA-B^*^08:01/HLA-B^*^35:01 donors also recognized significantly fewer HLA-mismatched EBV-LCLs than the corresponding T-cell populations isolated from HLA-B^*^08:01/HLA-B^*^13:02 heterozygous or HLA-B^*^08:02 homozygous donors (Figure 6B). These results show that the occurrence and broadness of allo-HLA cross-reactivity by virus-specific-specific T cells is influenced by the HLA background of the donors.

## Discussion

In this study, we demonstrated that 50% (83/164) of virus-specific T-cell populations contained T cells that cross-reacted against HLA-mismatched EBV-LCLs, in line with previous findings (11). We showed that the level of allo-HLA cross-reactivity is highly influenced by HLA restriction and not by the viral specificity of the virus-specific T-cell populations. HLA-B^*^08:01-restricted virus-specific T cells showed the highest frequencies and diversities of allo-HLA cross-reactivity compared to the HLA-A^*^01:01, HLA-A^*^02:01 or HLA-B^*^07:02-restricted virus-specific T-cell populations. Cross-reactivity against HLA-mismatched EBV-LCLs was shown to be mediated by recognition of allogeneic HLA molecules, which was confirmed by recognition of EBV^neg^ K562 cells transduced with specific HLA-class-I molecules, illustrating that the peptides presented by these allogeneic HLA molecules were not EBV or B-cell-derived. HLA-B^*^08:01-restricted virus-specific T cells showed a skewed pattern of recognition of a group of allogeneic HLA-B alleles, with HLA-B^*^35:01 being recognized most often. We demonstrated that cross-reactivities against multiple allogeneic HLA-class-I molecules by HLA-B^*^08:01-restricted EBV-EBNA3^QAK^-specific T cells could be mediated by single T-cell clones. Finally, heterozygosity for HLA-B^*^35:01, but not HLA-B^*^13:02 significantly reduced the degree of HLA cross-reactivity by HLA-B^*^08:01-restricted T cells, demonstrating that the HLA background of donors influences the off-target reactivity of virus-specific T cells.

Several groups have investigated whether the allo-HLA cross-reactive risk of virus-specific T cells could be predicted. In most of these studies, allo-HLA cross-reactive patterns could only be predicted when a T-cell population used a public TCR (14, 23, 24). Public T-cell populations could often be found by analysis of sub-populations of T cells expressing a single TCR-Vβ family. However, no pattern of allo-HLA cross-reactivity could be observed in our study, except for HLA-A^*^02:01-restricted EBV-LMP2^CLG^-specific T cells sorted for expression of TCR-Vβ5.1 (Figure 2C). Although virus-specific T cells often expressed the same TCR-Vβ family, differences in the Complementary Determining Region 3 (CDR3) or a different TCR-alpha chain could result in variation in the allo-HLA cross-reactivity patterns. Allo-HLA cross-reactivity can therefore not be predicted based on TCR-Vβ-family usage alone and may only result in clear patterns if the TCR-Vβ family consist of a public TCR (27).

Similar to other studies we observed that part of the allo-HLA cross-reactive T-cell populations showed recognition of HLA-mismatched EBV-LCLs, but no recognition of our panel of single HLA-class-I transduced K562 cells expressing 58% (*n* = 37/64) of the HLA-class-I molecules present in the EBV-LCL panel (11). The scope our current study was not to fully unravel the recognized allogeneic peptide in allo-HLA molecules. However, this may demonstrate that allo-HLA cross-reactive T cells do not solely recognize an household peptide in the context of allogeneic HLA, but potentially also lineage-specific peptide-allo-HLA cross-reactivity exists (28). Also recognition of HLA-class-II molecules by HLA-class-I-restricted CD8^pos^ virus-specific T cells has previously been described (11). However, in our study we did not see a correlation with the pattern of recognition against the EBV-LCL panel and the expression of specific HLA-class-II molecules. Therefore, HLA-class-II-restricted cross-reactivity was not further analyzed in depth in our current study.

Finding third-party donors with anti-viral T cells that are fully (HLA-class-I) matched to the recipient patients is probably difficult. When allo-HLA cross-reactive T cells targeting HLA alleles expressed on cells of the patient or (organ) donor are present in the virus-specific T-cell product, acute graft vs. host disease (GVHD) or graft rejection could occur. Strikingly, only a very low incidence of *de novo* acute GVHD or graft rejection has been observed in clinical trials analyzing the effect of adoptive T-cell therapy with third-party donor-derived products, either in the setting of HLA-mismatched stem cell transplantation or of solid organ transplantation (18, 29). It has therefore been assumed that third-party virus-specific T cells do not mediate GVHD or graft rejection (18). It is not clear whether, the observed absence of GVHD or graft rejection in these cases was the result of: (1) no expression of the particular mismatched HLA allele recognized by the transferred virus-specific T cells, (2) removal of the *in vitro* off-target (>10% cytotoxic) virus-specific T cells from the product prior to administration to the patient and/or selection of T-cell products that do not show *in vitro* allo-HLA reactivity (18), (3) extensive culturing of the virus-specific T cells prior to adoptive transfer, leading to senescence and impaired cytokine production (30), 4) weak adhesion molecule expression (i.e., ICAM-1) by the target organ (31), (5) biased production and administration of HLA-A^*^02:01-restricted virus-specific T cells with an intrinsic low risk of off-target toxicity, as shown in this study, (6) low T-cell numbers of cross-reactive virus-specific T cells administered and/or limited *in vivo* proliferation, or (7) rapid rejection of the virus-specific T cells (22).

Here, we demonstrated that around 40% of HLA-A^*^01:01, HLA-A^*^02:01, or HLA-B^*^07:02-restricted T-cell populations recognized one or more HLA-mismatched EBV-LCLs. For each T-cell population this recognition was found to be limited to only a few HLA-mismatched EBV-LCLs and could be attributed to recognition of one or a couple of allogeneic HLA alleles. The risk for accidentally mismatching for the particular allogeneic HLA allele(s) cross-recognized by the virus-specific T cells would be low, but studies do report cases of GVHD after infusion of virus-specific T cells derived from the SCT donor (32, 33) or derived from a third-party donor (33–35). Importantly, we found that HLA-B^*^08:01-restricted T cells isolated from donors that were homozygous for HLA-B^*^08:01 or heterozygous for HLA-B^*^08:01 and a specific HLA-B allele (e.g., HLA-B^*^13:02) showed abundant allo-HLA cross-reactivity *in vitro* and are therefore likely to cause graft rejection or GVHD *in vivo*. Since in the majority of studies so far, the adoptive transfer of third-party donor-derived virus-specific T cells was focused on HLA-A^*^02:01- and/or HLA-B^*^07:02-restricted virus-specific T cells (36), the effect of HLA-B^*^08:01-restricted virus-specific T cells has not been extensively studied (37). Our results on the higher incidence of HLA-cross-reactivity by HLA-B^*^08:01-restricted compared to HLA-A^*^01:01, HLA-A^*^02:01, or HLA-B^*^07:02-restricted virus-specific T cells may have important value for the design of future clinical trials. Since the specificity did not contribute to the allo-HLA cross-reactivity, these results have also important value for third-party derived CAR-T cell therapies or in the field of organ transplantations. Intriguingly, studies in the field of organ transplantations show a significant increase of acute graft rejections in recipients that express HLA-B^*^08:01, HLA-C^*^07:01, and HLA-DRB1^*^03:01 (38, 39). These three HLA molecules are part of a common haplotype (40) and the homozygous donors used in our study have the same haplotype, suggesting that these rejections are mediated by HLA-B^*^08:01-restricted T cells. Altogether, these results imply that the HLA background of the donor is important for the broadness of the allo-HLA cross-reactivity. Therefore, the most compatible HLA background of the donor should be aimed for and homozygous donors should not be used despite the lower chance of rejection.

Since we only analyzed virus-specific T cells restricted to four different HLA molecules, it remains unclear whether T cells with another HLA restriction could show similar reactivity patterns as HLA-B^*^08:01-restricted T cells. However, we hypothesize that these findings might only be restricted to a few HLA molecules since the peptidome of HLA-B^*^08:01 shows an unique pattern, that is specific for only HLA-B^*^08:01 and HLA-B^*^08:02. Based on binding data and sequence information, Sidney J. et al. classified the majority of HLA-B molecules into 9 super families (41). We hypothesized that super families with only a few HLA-B alleles, have unique peptidomes and T cells with this specific HLA background are likely to be cross-reactive against HLA molecules from other HLA super families, since negative thymic selection for these peptide-HLA complexes has not taken place. In the present study, virus-specific T cells isolated from donors that expressed HLA-B^*^08:01 and HLA-B^*^35:01 proved to be less allo-HLA cross-reactive than those from donors that were homozygous for HLA-B^*^08:01 or heterozygous for HLA-B^*^08:01 and HLA-B^*^13:02. We hypothesize that HLA-B^*^35:01 may elicit thymic negative selection for all HLA molecules present in the B07 superfamily to which it belongs (e.g., HLA-B^*^07:02, HLA-B^*^35:03, HLA-B^*^42:01). Being heterozygous for any of the HLA molecules from this B07 superfamily would then presumably result in the same outcome as heterozygosity for HLA-B^*^35:01. HLA-B^*^13:02 could not be assigned to a particular HLA superfamily (41), possibly explaining why it did little to the level of allo-HLA cross-reactivity of the HLA-B^*^08:01-restricted repertoire in our study. Therefore, if full matching for HLA-B is not possible, we propose that donors should be used that express HLA-B molecules that are part of different superfamilies to reduce the chance for a broad off-target toxicity in clinical application of third-party donor-derived T-cell products.

Altogether, our results indicate that selection of virus-specific T-cells with specific HLA restrictions and donors with specific HLA backgrounds may decrease the risk of developing GvHD or (organ) graft rejection after infusion of third-party donor-derived virus-specific T cells into patients with uncontrolled viral reactivation. Ideally, if complete HLA-class-I matching is not feasible, donor and recipient should at least be fully matched for HLA-B or matched for HLA-B alleles from the same HLA-B superfamily. Mismatching of HLA-B alleles that are unclassified should be avoided, because the peptides presented by these HLA-molecules are unique and could mediate allo-HLA cross-reactivity.

## Data Availability Statement

The original contributions presented in the study are included in Supplementary Figure 1, further inquiries can be directed to the corresponding author/s.

## Author Contributions

WH, DL, LM, and LH, performed experiments. WH analyzed results and made the figures. WH, JF, DA, and IJ designed the research and wrote the paper. All authors contributed to the article and approved the submitted version.

## Conflict of Interest

The authors declare that the research was conducted in the absence of any commercial or financial relationships that could be construed as a potential conflict of interest.
