# Supplementary material for: Magnitude of Off-Target Allo-HLA Reactivity by Third-Party Donor-Derived Virus-Specific T Cells Is Dictated by HLA-Restriction
Source: Front Immunol. 2021 Mar 29;12:630440. doi: 10.3389/fimmu.2021.630440 (PMC8039299; doi:10.3389/fimmu.2021.630440)
Supplement: Supplementary file 1 [file Data_Sheet_1.DOCX]

**Magnitude of Off-Target Allo-HLA Reactivity by Third Party Donor-derived Virus-Specific T Cells is Dictated by HLA-Restriction**

Huisman W ^1,2^, Leboux D.A.T^1^, van der Maarel L.E^1^, Hageman L^1^, Amsen D^2^, Falkenburg J.H.F^1^, Jedema I^1^

*^1^Department of Hematology, Leiden University Medical Center, The Netherlands; ^2^Department of Hematopoiesis, Sanquin Research and Landsteiner Laboratory for Blood Cell Research, Amsterdam, the Netherlands*

**Online Supplementary Appendix**

**Material and Methods**

**Generation of peptide-MHC complexes to isolate virus-specific T cells**

All viral peptides were synthesized in-house using standard Fmoc chemistry. Recombinant HLA-A*01:01, HLA-A*02:01, HLA-B*07:02 and HLA-B*08:01 heavy chain and human β2m light chain were in-house produced in Escherichia coli. MHC-class-I refolding was performed as previously described with minor modifications^1^. Major histocompatibility complex (MHC)-class-I molecules were purified by gel-filtration using HPLC. Peptide-MHC(pMHC) tetramers were generated by labeling biotinylated pMHC-monomers with streptavidin-coupled phycoerythrin (PE; Invitrogen, Carlsbad, USA), allophycocyanin (APC, Invitrogen), brilliant violet 421 (BV421, Becton Dickinson (BD), Franklin Lakes, USA), brilliant violet 510 (BV510, BD) or peridinin-chlorophyll-protein complex (PerCP, Invitrogen). Complexes were stored at 4 °C. Formation of stable pMHC-monomers was performed using UVexchange technology^2^ and according to a previously described protocol^3^.

**Isolation and expansion of virus-specific T cells**

PBMCs from healthy donors were first incubated with pMHC-tetramer complexes for 30 min at 4°C followed by PerCP-labeled CD8 (BD) and fluorescein isothiocyanate-labeled (FITC) CD4 and CD14 (BD) antibodies at 4°C for 30 min. Single pMHC-tetramer^pos^ virus-specific T cells were first specifically expanded, in the presence of 10^-7^ M of the specific peptide in T-cell medium: Iscove’s Modified Dulbecco’s Medium (IMDM; Lonza, Verviers, Belgium) containing 5% heat-inactivated fetal bovine serum (FBS; Invitrogen), 5% heat-inactivated human serum (ABOS; Sanquin Reagents, Amsterdam, The Netherlands), 100 U/mL penicillin (Lonza), 100 µg/mL streptavidin (Lonza) , 2.7mM L-glutamine (Lonza) 100 IU IL-2/ml (Chiron, Emeryville, USA) and with 5-fold 35 Gy irradiated autologous PBMCs as feeder cells. Specific stimulation was first performed to enrich for only pMHC-tetramer^pos^ T cells. Subsequently, pMHC-tetramer^pos^ T-cell populations were further non-specifically expanded using the aforementioned feeder mixture with 0.8μg/ml phytohemagglutinin (PHA; Oxoid Limited, Basingstoke, UK) instead of specific peptide.

**Selection and generating stimulator cells for functional analyses**

Constructs encoding different HLA-class-I sequences were coupled to an IRES sequence with a truncated form of the nerve growth factor receptor (tNGFR) serving as marker gene and were cloned into LZRS plasmids. Constructs were verified using reverse transcriptase polymerase chain reactions (RT-PCR) and Sanger sequencing. As a control, tNFGR only was cloned into an LZRS plasmid (mock). Retroviral transduction was performed as previously described^4^. K562 wildtype cell-lines were transferred to wells containing stable retroviral particles, generated using a puromycin selected stable ϕ-NX-A packaging cell line, and incubated for 24 hrs at 37°C^5^. For FACSorting, cells were stained with PE-labelled NGFR antibodies (NGFR(CD271); BD/Pharmingen) for 30 min at 4°C. After sorting, single HLA-class-I transduced K562 cells were analyzed for HLA-class-I expression using FITC-labelled HLA-ABC antibodies (Serotec, Hercules, USA) and PE-labelled NGFR(BD). Single HLA-class-I transduced K562 cell-lines were verified using RT-PCR and Sanger sequencing

**Cytotoxicity assay**

Cytotoxicity was determined by 51-chromium (^51^Cr)-release assay. Virus-specific T-cell populations were added to (10:1, E:T ratio) ^51^Cr-labeled EBV-LCLs and single HLA-class-I transduced K562 cells for 4 hrs at 37°C in the same IMDM medium used for cytokine production assays. ^51^Cr release was measured on a γ-counter. Spontaneous ^51^Cr release of the target cells was determined in medium alone, and maximum ^51^Cr release was determined by adding Triton (1%; Sigma, Saint louis, USA). Percentages of specific lysis were determined by the following calculation: ((experimental ^51^Cr release – averaged spontaneous ^51^Cr release) / (averaged maximal ^51^Cr release – averaged spontaneous ^51^Cr release)) x 100. Values for specific ^51^Cr lysis represent the mean plus and minus standard deviation of triplicate wells. Spontaneous and maximum release represents the mean of sextuplicate wells.

**Results**

**Supplementary table 1. Peptide-MHC-tetramers used for the isolation of virus-specific T-cell populations.**

| **Virus** | **Antigen** | **HLA** | **Peptide** |
| --- | --- | --- | --- |
| CMV | pp50 | HLA-A*01:01 | VTEHDTLLY |
|  | pp65 | HLA-A*01:01 | YSEHPTFTSQY |
|  | pp65 | HLA-A*02:01 | NLVPMVATV |
|  | IE-1 | HLA-A*02:01 | VLEETSVML |
|  | pp65 | HLA-B*07:02 | TPRVTGGGAM |
|  | pp65 | HLA-B*07:02 | RPHERNGFTVL |
|  | IE-1 | HLA-B*08:01 | ELRRKMMYM |
|  | IE-1 | HLA-B*08:01 | QIKVRVDMV |
| EBV | LMP2 | HLA-A*01:01 | ESEERPPTPY |
|  | LMP2 | HLA-A*02:01 | FLYALALLL |
|  | LMP2 | HLA-A*02:01 | CLGGLLTMV |
|  | EBNA3C | HLA-A*02:01 | LLDFVRFMGV |
|  | BMLF1 | HLA-A*02:01 | GLCTLVAML |
|  | BRLF1 | HLA-A*02:01 | YVLDHLIVV |
|  | EBNA3A | HLA-B*07:02 | RPPIFIRRL |
|  | BZLF1 | HLA-B*08:01 | RAKFKQLL |
|  | EBNA3A | HLA-B*08:01 | FLRGRAYGL |
|  | EBNA3A | HLA-B*08:01 | QAKWRLQTL |
| AdV | HEXON | HLA-A*01:01 | TDLGQNLLY |
|  | E1A | HLA-A*02:01 | LLDQLIEEV |
|  | HEXON | HLA-B*07:02 | KPYSGTAYNAL |

CMV, Cytomegalovirus; EBV, Epstein-Barr virus; AdV, Adenovirus

**Supplementary table 2. Single HLA-class-I transduced K562 cell-lines.**

| **HLA-A** | **HLA-B** | **HLA-C** |
| --- | --- | --- |
| 01:01 | 07:02 | 01:02 |
| 02:01 | 08:01 | 03:03 |
| 02:05 | 13:02 | 05:01 |
| 02:60 | 14:02 | 06:02 |
| 03:01 | 15:01 | 07:01 |
| 11:01 | 15:02 | 07:02 |
| 23:01 | 27:05 | 14:02 |
| 24:02 | 35:01 | 16:01 |
| 30:02 | 35:03 |  |
| 32:01 | 35:231 |  |
| 33:01 | 38:01 |  |
| 68:01 | 39:01 |  |
|  | 40:01 |  |
|  | 41:01 |  |
|  | 44:02 |  |
|  | 44:03 |  |
|  | 51:01 |  |
|  | 52:01 |  |
|  | 55:01 |  |
|  | 57:01 |  |
| *n=12* | *n=20* | *n=8* |

The K562 panel was composed of HLA-deficient K562 cells retrovirally transduced with constructs encoding specific HLA-class-I alleles. All HLA-constructs and transduced K562 cell-lines were checked by Sanger sequencing.


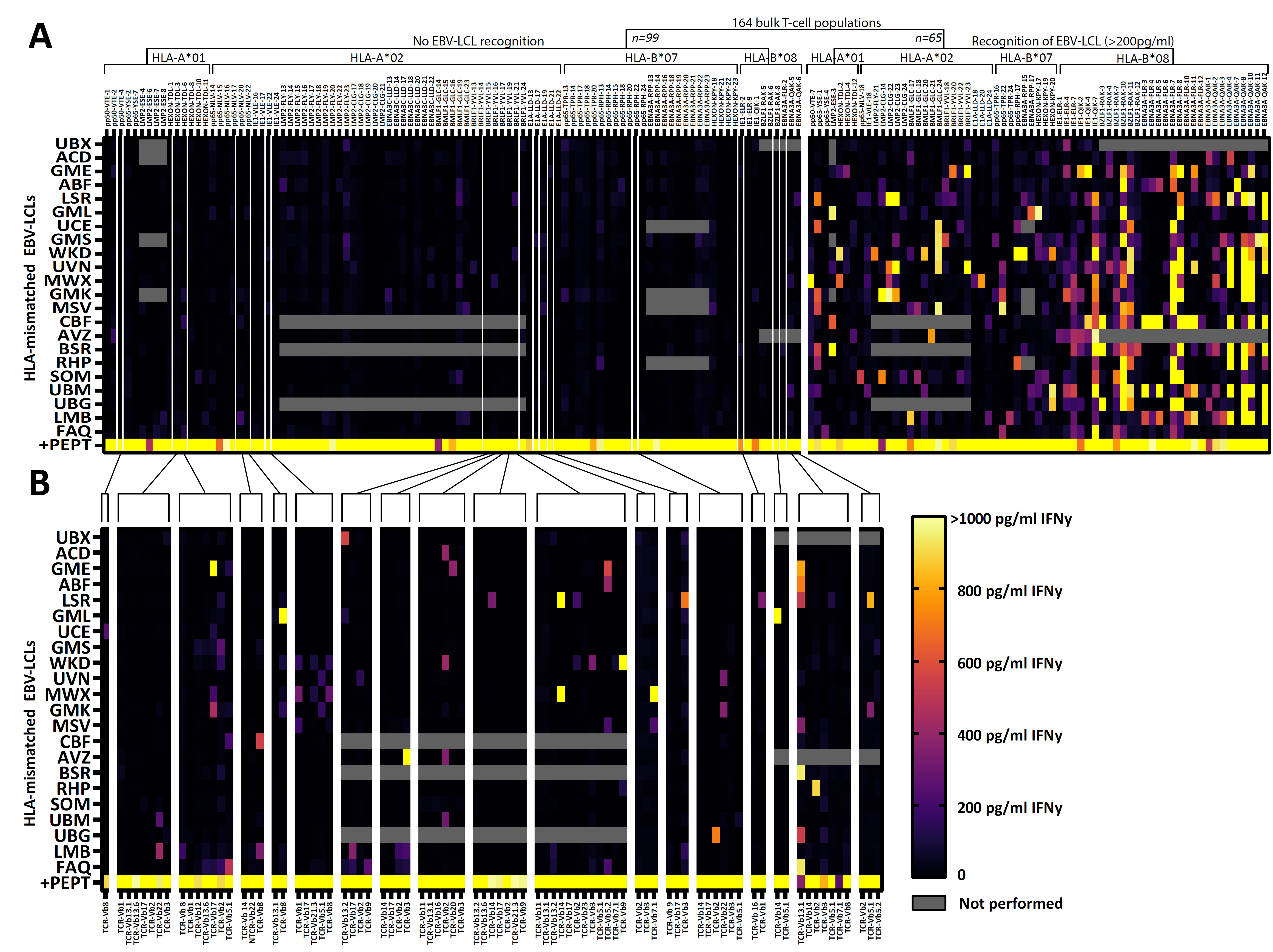


**Supplementary Figure 1. Virus-specific T-cell populations show profound and diverse cross-reactivity against a panel of HLA-mismatched EBV-LCLs.** Virus-specific T-cell populations were stimulated with a panel of HLA-mismatched EBV-LCLs for 16hrs and IFNγ production was measured by ELISA. Reactivity was defined as production of >200pg/ml IFNγ. EBV-specific T-cell populations were tested only against those HLA-mismatched EBV-LCLs that did not express the specific restriction molecule of the viral specificity of those T cells. EBV-LCLs from donor OBB exogenously loaded with 10^-6^M of the respective viral peptide were used as positive control. **A)** The results of cross-reactivities against HLA-mismatched EBV-LCLs mediated by bulk virus-specific T cells is shown and divided in bulk virus-specific T-cell populations that did not show any cross-reactivity (<200pg/ml IFNγ, left) and bulk virus-specific T-cell populations that showed profound and diverse cross-reactivity against the HLA-mismatched EBV-LCLs (right). The different bulk virus-specific T-cell populations are shown on the x-axis clustered per HLA-restriction and their specificity and origin are shown as protein-peptide-donorID. **B)** Shown are virus-specific T cells that were sorted for expression of a single TCR-Vβ family and that were stimulated with HLA-mismatched EBV-LCLs and demonstrated recognition of HLA-mismatched EBV-LCLs, while the original bulk virus-specific T-cell population did not show any recognition. The x-axis shows the different TCR-Vβ family expression of the sorted T-cell populations.

TCR, T-cell Receptor. Vβ, Variable Beta Chain.


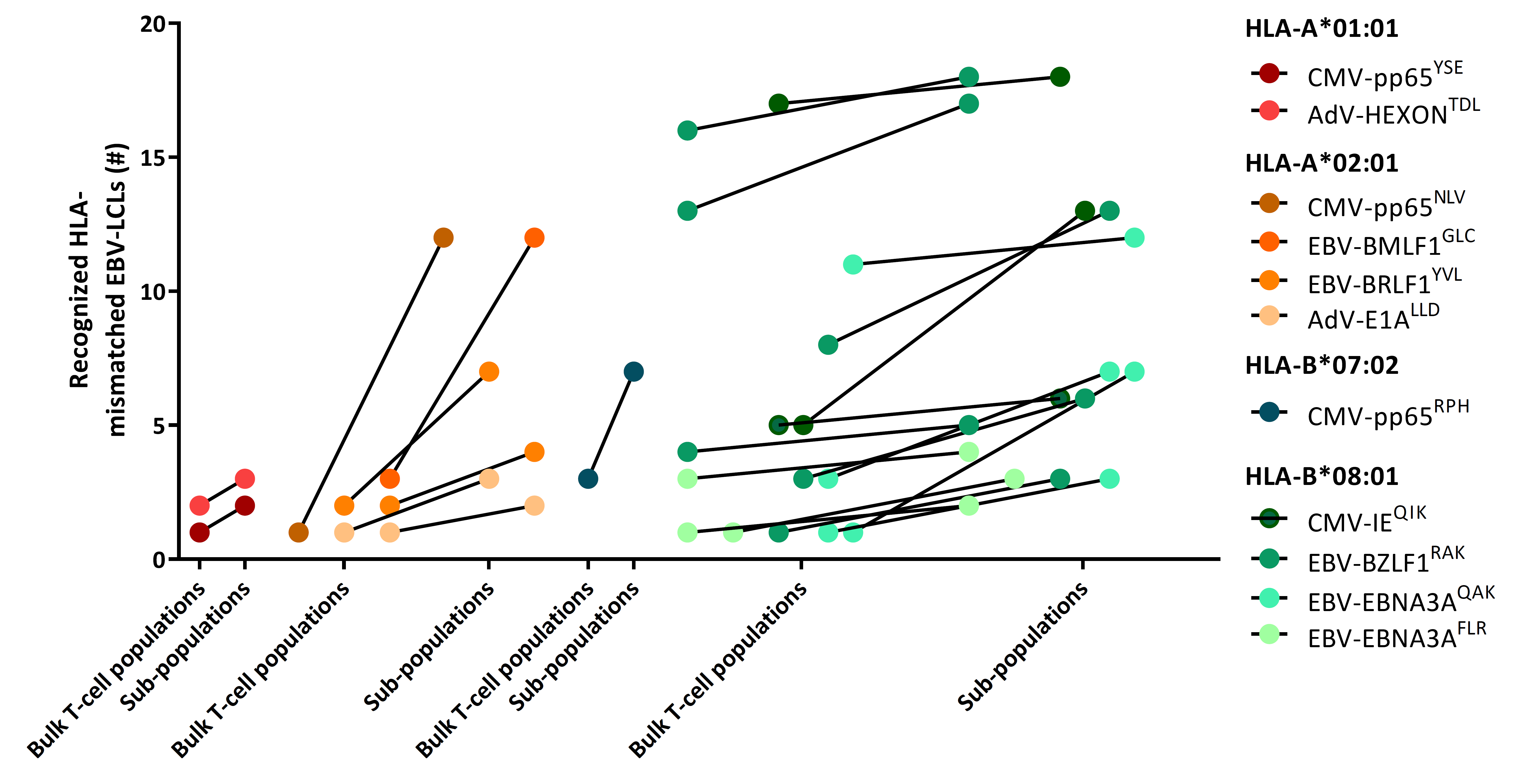


**Supplementary Figure 2. Recognition of additional HLA-mismatched EBV-LCLs when sub-populations expressing single TCR-Vβ families were included in the analysis.** T-cell populations expressing a single TCR-Vβ family were sorted from the bulk virus-specific T-cell populations and stimulated with a panel of HLA-mismatched EBV-LCLs for 16hrs and IFNγ production was measured by ELISA. Recognition of HLA-mismatched EBV-LCLs was defined as production of >200pg/ml IFNγ by the virus-specific T cells. Shown are 25 bulk T-cell populations from which sub-populations expressing single TCR-Vβ families were sorted that demonstrated additional recognition of HLA-mismatched EBV-LCLs.

**Supplementary Figure 3. Cross-reactivity of HLA-B*07:02-restricted CMV-pp65^RPH^ T cells.** The CMV-pp65^RPH^-specific T-cell population was stimulated with a panel of K562 cells transduced with HLA molecules to investigate which other HLA-molecules were recognized based on the reactivities seen against the EBV-LCL panel. K562 cells transduced with HLA-B*07:02 exogenously loaded with 10^-6^M of the respective viral peptide was used as positive control.

**Supplementary Figure 4. Allo-HLA cross-reactive virus-specific T cells lyse HLA mismatched target cells.** To investigate whether the allo-HLA cross-reactivity of virus-specific T cells also results in cytotoxicity, virus-specific T-cell lines were tested in cytotoxicity assays against HLA-mismatched EBV-LCLs and single HLA-class-I transduced K562 cell-lines. Two representative examples are shown. **A)** The allo-HLA-B*27:05/HLA-B*40:01 cross-reactive HLA-B*07:02-restricted TCR-Vβ5.1 sorted CMV-pp65^RPH-^specific T-cell population derived from donor #17 was tested against 4 HLA-mismatched EBV-LCLs and 5 single HLA-class-I transduced K562 cell-lines **B)** The allo-HLA-A*68/HLA-B*07:02 cross-reactive HLA-A*01:01-restricted CMV-pp65^YSE^-specific T-cell population derived from donor #1 was tested against 4 HLA-mismatched EBV-LCLs and 5 single HLA transduced K562 cell lines (HLA-B*14:02, HLA-B*52:01, HLA-A*68:01, HLA-B*07:02 and peptide “YSE” pulsed HLA-A*01:01).

Shown are means with standard deviations of 1 experiment carried out in triplicate, with an effector: target (E:T) ratio of 10:1.

TCR, T-cell Receptor. Vβ, Variable Beta Chain


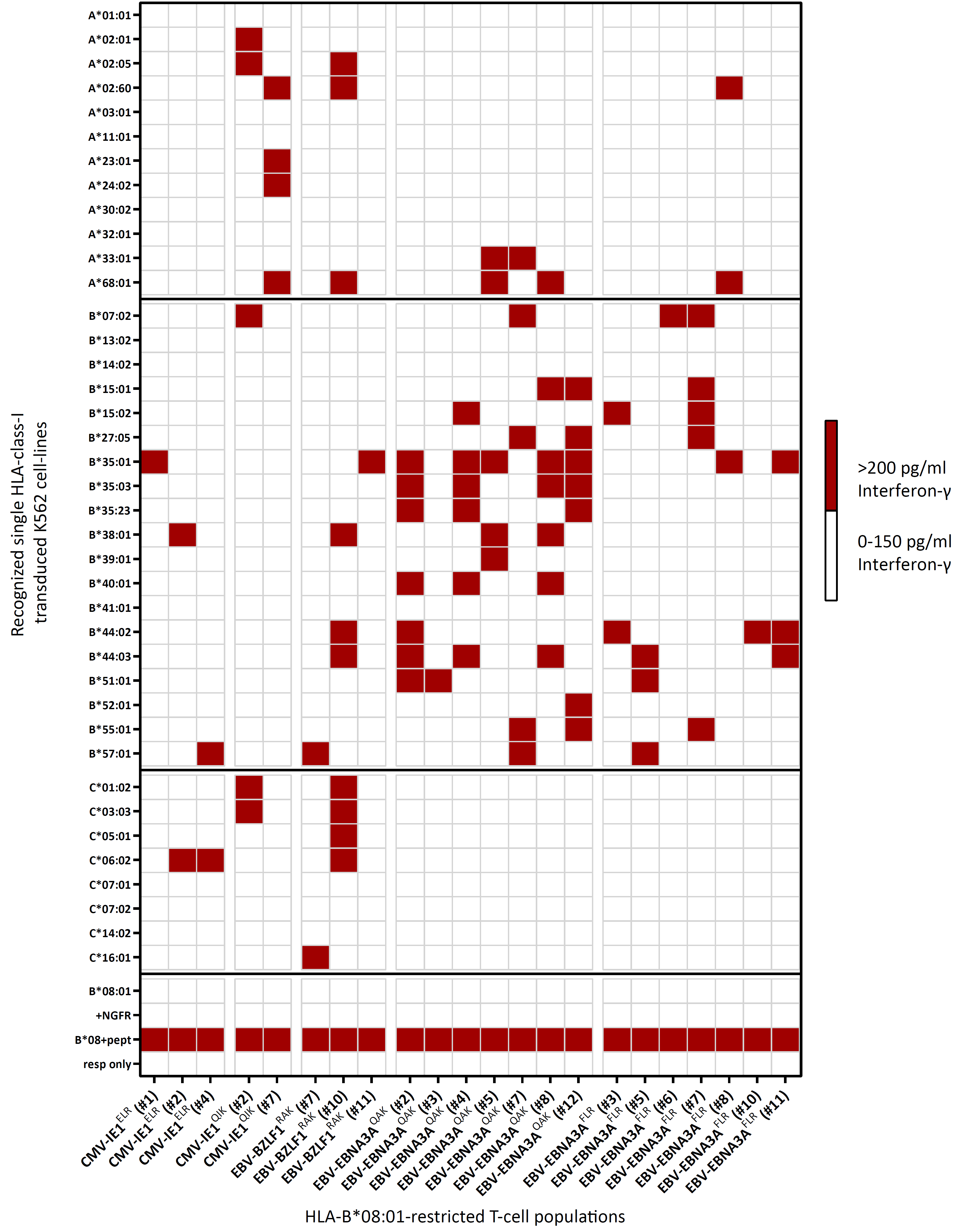
**Supplementary figure 5. Skewed cross-reactivity against certain allogeneic HLA-B alleles by HLA-B*08:01-restricted virus-specific T cells.** HLA-B*08:01-restricted T-cell populations (n=22) with no clear recognition pattern when tested against the HLA-mismatched EBV-LCL panel, were stimulated with a panel of single HLA-class-I transduced K562 cell-lines (n=40) for 16hrs and IFNγ production was measured by ELISA to analyze which HLA molecules were being recognized. Reactivity was defined as production of >200pg/ml IFNγ. Shown are the specific allogeneic HLA-A, B or C molecules (y-axis) recognized by the HLA-B*08:01-restricted T-cell populations directed against different CMV or EBV epitopes (x-axis). The numbers between brackets represent the identification numbers of the donors from which the respective T-cell population originate.

**References**

1. Garboczi DN, Hung DT, Wiley DC. HLA-A2-peptide complexes: refolding and crystallization of molecules expressed in Escherichia coli and complexed with single antigenic peptides. *Proc Natl Acad Sci U S A.* 1992;89(8):3429-3433.

2. Rodenko B, Toebes M, Hadrup SR, et al. Generation of peptide-MHC class I complexes through UV-mediated ligand exchange. *Nat Protoc.* 2006;1(3):1120-1132.

3. Eijsink C, Kester MG, Franke ME, et al. Rapid assessment of the antigenic integrity of tetrameric HLA complexes by human monoclonal HLA antibodies. *J Immunol Methods.* 2006;315(1-2):153-161.

4. Heemskerk MH, Hoogeboom M, de Paus RA, et al. Redirection of antileukemic reactivity of peripheral T lymphocytes using gene transfer of minor histocompatibility antigen HA-2-specific T-cell receptor complexes expressing a conserved alpha joining region. *Blood.* 2003;102(10):3530-3540.

5. D'Orsogna LJ, van der Meer-Prins EM, Zoet YM, Roelen DL, Doxiadis, II, Claas FH. Detection of allo-HLA cross-reactivity by virus-specific memory T-cell clones using single HLA-transfected K562 cells. *Methods in molecular biology (Clifton, NJ).* 2012;882:339-349.
